# Timing of Intrapartum Antibiotics at Caesarean Section and Risk of Asthma, Eczema and Allergic Rhinitis: Results From a Natural Experiment

**DOI:** 10.1111/1471-0528.70083

**Published:** 2025-11-14

**Authors:** Lucy Pembrey, Gillian Santorelli, Sergio Souza da Cunha, Sam Oddie, Emily S. Petherick, Neil Pearce, Amy Hough, John Wright

**Affiliations:** ^1^ Department of Medical Statistics London School of Hygiene & Tropical Medicine London UK; ^2^ Bradford Institute for Health Research Bradford Teaching Hospitals NHS Foundation Trust Bradford UK; ^3^ School of Sport, Exercise and Health Sciences Loughborough University Loughborough UK; ^4^ National Institute for Health Research (NIHR) Leicester Biomedical Research Centre University Hospitals of Leicester NHS Trust and University of Leicester Leicester UK

**Keywords:** allergic rhinitis, asthma, caesarean section, eczema, prophylactic antibiotics

## Abstract

**Objective:**

To investigate whether the risk of asthma, eczema and allergic rhinitis at 5 years in children born by caesarean section (CS) differs by the timing of antibiotic administration to the mother (pre‐incision vs. post‐cord clamping).

**Design:**

Natural experiment using birth cohort data.

**Setting:**

UK single centre population‐based birth cohort.

**Participants:**

3013 liveborn children delivered by CS, from the Born in Bradford (BiB) cohort born 2007–2011 and BiB's Better Start (BiBBS) cohort born 2016–2019. Only the first‐born of multiple births was included.

**Main Outcome Measures:**

Risk of asthma, atopic eczema and allergic rhinitis at age 5 years.

**Results:**

Among 3013 children, 579 (19.2%) were exposed to pre‐incision antibiotics. At 5 years, 272 (9.0%) children had asthma, 672 (22.3%) had eczema and 180 (6.0%) had allergic rhinitis. There was no evidence of an increased risk of asthma (adjusted risk ratio [aRR] 1.01, 95% CI 0.56, 1.83; adjusted risk difference [aRD] 0.08 per 100, 95% CI −5.28, 5.44), eczema (aRR 0.96, 95% CI 0.68, 1.35; aRD −1.01 per 100, 95% CI −8.65, 6.63) or allergic rhinitis (aRR 1.16, 95% CI 0.59, 2.28; aRD 0.88 per 100, 95% CI −3.17, 4.92) at 5 years in children exposed to pre‐incision antibiotics versus post‐cord clamping.

**Conclusions:**

This study found no evidence of an association between pre‐incision antibiotics for CS and increased risk of asthma, eczema or allergic rhinitis by age 5. These findings contribute to the evidence base informing the optimum timing of maternal antibiotic prophylaxis for CS, balancing risks and benefits to the mother and her child.

## 1 Introduction

There has been considerable controversy about the role of antibiotic use in pregnancy and early childhood and the subsequent risk of asthma, eczema and allergic rhinitis [1, 2]. Several recent studies have found results consistent with early life antibiotic exposure as a cause of asthma [3, 4], with smaller effects of late‐prenatal antibiotic exposure [3].

Women who undergo caesarean section (CS) receive intrapartum antibiotic prophylaxis to prevent post‐caesarean surgical site infection. Historically, these antibiotics were administered to the mother after the umbilical cord was clamped. However, international guidelines now recommend that antibiotics are given to the mother before the incision for CS, as there is good evidence that this reduces the risk of wound infections [5, 6]. The UK NICE guidance changed in 2011 [7, 8], although some hospitals, including Bradford Royal Infirmary, began administering pre‐incision antibiotics prior to this, responding to evidence of reductions in post‐operative infections [9, 10]. This change in the timing of antibiotic administration means that babies born by CS will be exposed to a dose of antibiotics just before birth which could affect their risk of asthma and allergic disease, via an impact on the gut microbiota that can affect the development of immune responses [11, 12, 13].

The Born in Bradford (BiB) study provided the opportunity of a natural experiment to compare the risk of asthma and allergic disease in children born by CS and exposed to antibiotics with those born by CS but not exposed to antibiotics prior to delivery. At Bradford Royal Infirmary the change in timing of antibiotics administration occurred gradually during the period 2009–2010. The exposure status of each child was determined by the policy in place at the time of birth rather than any individual maternal or child characteristics, so potential biases and confounding, which have been an issue in other studies of antibiotic exposure and asthma/allergic disease, are avoided [14, 15]. BiB combines research data with linkage to primary and secondary care records, and importantly had access to paper maternity records for earlier births to determine individual exposure status, which was not available in routine electronic health records (EHR).

This study aims to investigate whether the risk of asthma, eczema and allergic rhinitis at 5 years in children born by CS differs by the timing of antibiotic administration to the mother during delivery (pre‐incision vs. post‐cord clamping). The secondary outcomes are maternal surgical site infections following CS, and wheeze, eczema and allergic rhinitis at 2 years (to assess any shorter‐term effects).

## 2 Methods

### 2.1 Participants and Setting

Born in Bradford (BiB) is an internationally renowned research programme tracking the health and wellbeing of over 60 000 participants in Bradford, UK. Between 2007 and 2011, the BiB Family cohort enrolled 12 453 pregnant women, resulting in 13 858 births. The cohort is broadly representative of the Bradford population [16]. Born in Bradford Better Start (BiBBS) is an interventional birth cohort within BiB, comprising over 5000 babies born between 2016 and 2024 in three inner‐city, socioeconomically deprived areas of Bradford [17]. At the time of recruitment for both BiB and BiBBS, pregnant women completed an administered questionnaire and consented to the linkage of their clinical records from maternity systems, primary and secondary care, and community health data. Linkage to these datasets was successful in over 98% of participants.

Inclusion criteria: The study included liveborn children from the BiB cohort (born 2007–2011) and BiBBS children born between 2016 and 2019, ensuring they had reached the age of 5 years for outcome assessment. If a mother had multiple singleton pregnancies during the recruitment period, all her children were included. For multiple births, only the first‐born child was included, as children from multiple births share exposure status.

Exclusion criteria: Children born in a hospital other than Bradford Royal Infirmary (BRI) were not eligible, as information on type of birth and antibiotic administration was not available. Mothers or children who withdrew from the study or died before age 5 were not included.

The study protocol was amended from its original focus on BiB children to include BiBBS participants to increase the sample size of children exposed to pre‐incision antibiotics (https://fundingawards.nihr.ac.uk/award/16/150/06).

### 2.2 Exposure

For BiB participants, the timing of prophylactic antibiotic administration was determined through a detailed review of paper maternity records. Information was collected on the time of birth, the timing and type of antibiotics, and the length of post‐partum hospital stay. A standard data entry form was completed by a trained researcher for each eligible baby in the study, using a secure online data entry system which linked with the BiB database to identify each baby using the hospital number and date of birth.

A detailed protocol to determine the timing of antibiotic administration from paper records ensured the process was as objective as possible. For example, the sequence of medications documented on anaesthetic charts was used rather than just relying on the time of administration in relation to the time of birth, which can be difficult to determine accurately from some records. In cases where there were doubts about the timing, a consultant obstetrician or neonatologist at BRI was consulted.

As children in the BiBBS cohort were born since 2016, it is highly likely that those born by CS would have been exposed to pre‐incision antibiotics and this was confirmed by review of a random sample of 59 maternal records.

### 2.3 Outcomes

1. Children with asthma, atopic eczema and allergic rhinitis were identified through linked primary care diagnosis and prescription data. Outcomes at five (and two) years were defined as follows [18]:

Asthma at 5 years: a diagnosis of asthma between 3 and 5 years of age.

Wheeze at 2 years: a diagnosis of wheeze or asthma between birth and 2 years of age.

Eczema: a diagnosis of eczema between 1 and 5 (1 and 2) years of age and at least two prescriptions of eczema‐related treatment within 90 days before or 365 days after the first recorded eczema diagnosis (requirement for prescription only used for outcome at 5 years).

Allergic rhinitis: a diagnosis of allergic rhinitis or allergic conjunctivitis by 5 (2) years of age.

2 Maternal surgical site infections following CS were identified from linked primary and secondary care records. Cases were identified using primary care records where:
  - Read code XaC10 (infection of caesarean section wound following delivery) is recorded OR.
  - Any Read code listed in Table S1 if the infection occurred between 0 and 60 days after CS AND there were no other surgical procedures performed within 30 days before or after birth (data obtained from secondary care records).

Cases were identified using secondary care records where:

- ICD‐10 code O86 (Infection of obstetric surgical wound) is recorded within 60 days of CS.
- Any other surgical procedures performed up to 30 days before or after birth were reviewed. Procedures that were unclear or could potentially have been performed during the CS were excluded.

### 2.4 Confounders

Whether a mother received prophylactic antibiotics pre‐incision or post‐cord clamping was determined by the clinical policy in place at the time. As such, the timing of antibiotic administration was associated with the period during which the birth occurred, rather than with any individual maternal characteristics. Since the BiB and BiBBS cohorts span different time periods and exhibit some variation in key maternal characteristics, such as ethnicity, month and year of birth were included a priori in the analysis.

We constructed a directed acyclic graph (DAG) using existing literature and clinical understanding of the relationship between antibiotic exposure and the primary outcomes (Figure S1). We identified several potential confounders including sex, maternal history of asthma and allergic disease, highest maternal educational attainment (as a measure of socioeconomic status), number of children aged under 16 in the household, smoking during pregnancy, ethnicity, breastfeeding and other antibiotics prescriptions to mothers and children. These data were obtained from study questionnaires and linked primary and secondary care data. The absence of diagnostic or prescription information for asthma, eczema and allergic rhinitis in children and mothers, and antibiotics prescriptions was assumed to indicate the absence of these conditions.

### 2.5 Statistical Analysis

Statistical analysis was conducted using Stata, Version 17. Maternal and child characteristics were summarised using mean (SD) for continuous variables and frequency (%) for categorical variables. Poisson regression models were used to estimate risk ratios with 95% confidence intervals (CI) for the association between pre‐incision antibiotic exposure and each outcome, adjusting for the confounders described above. To account for clustering of multiple children from the same mother, we applied a clustered sandwich estimator, ensuring robust standard errors that accounted for within‐cluster correlation. Missing data for educational attainment, number of children in the household, smoking status and breastfeeding were addressed using multiple imputation via chained equations, generating 20 imputed datasets. The mimrgns command was used to calculate adjusted risk differences at 5 years [19]. Two sensitivity analyses were conducted: (1) using a complete case analysis, (2) excluding children whose 0–2 year window overlapped major COVID‐19 restrictions, to address altered infection/prescribing patterns.

### 2.6 Study Power

To detect a risk ratio of 1.5 for outcomes at age 5 for exposure to pre‐incision antibiotics, our study had 88.7% power for asthma, 99.0% power for eczema and 73.5% power for allergic rhinitis.

### 2.7 Patient and Public Involvement

We met with the BiB Parent Governor Group, comprising parents and carers of children enrolled in BiB (usually 8–12 families represented), four times to discuss this study: first at the design stage before the funding application was submitted, 6 months after the start of the study, a year later and at the end of the study. As the study did not involve direct contact with participants, the main role of the BiB Parent Governor Group was to advise us on communicating the aims and findings of the study to BiB families and the general public.

### 2.8 Ethical Approval

This study has Health Research Authority (HRA) and Health and Care Research Wales (HCRW) approval (ref: 238908). The Born in Bradford project has ethical approval from the Bradford Research Ethics Committee (ref: 07/H1302/112). Women recruited to BiB and BiBBS gave informed consent for their participation and on behalf of their children.

## 3 Results

**FIGURE 1:**
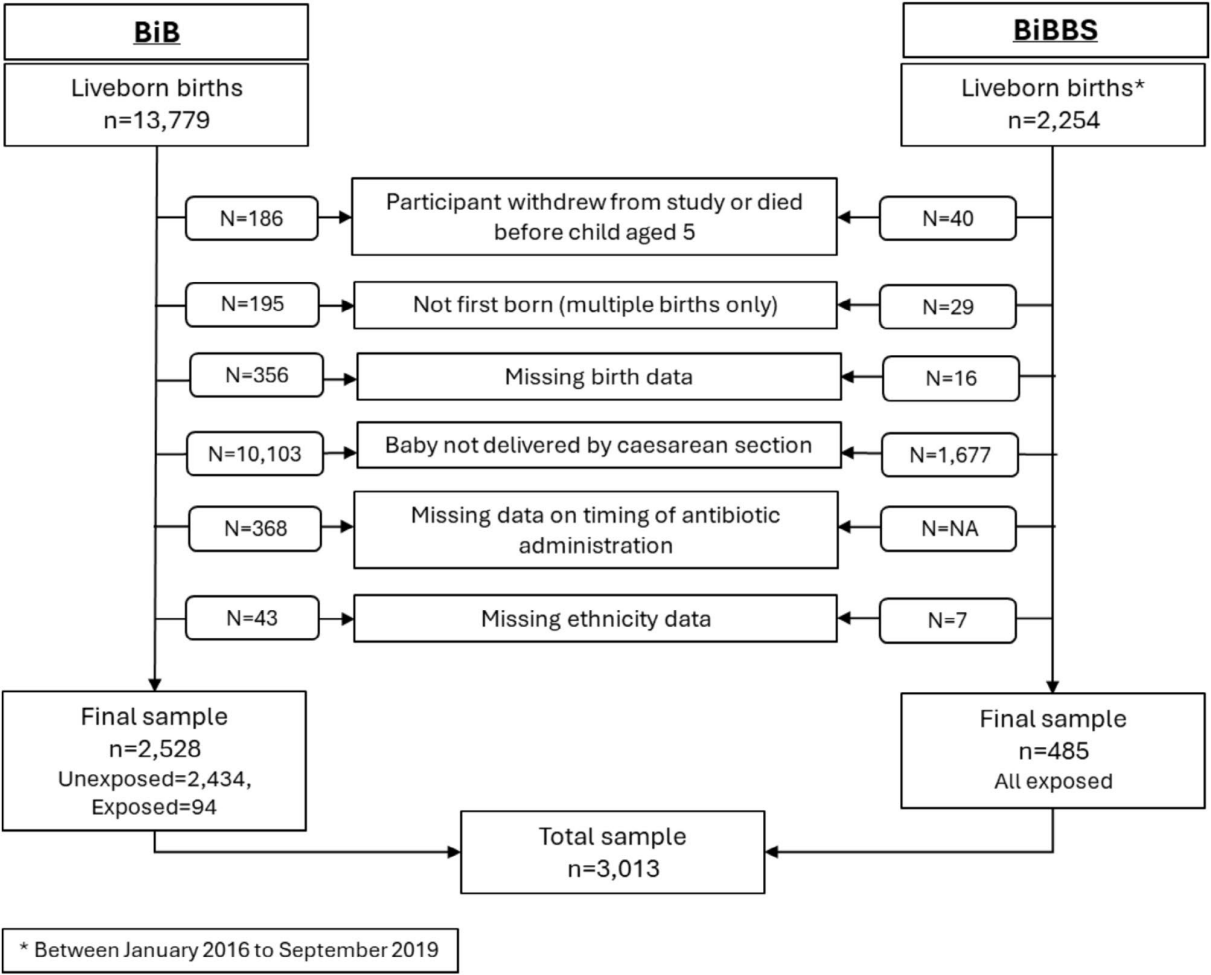
Sample flowchart.

A total of 3013 children were included (Figure 1), of whom 579 (19.2%) were exposed to pre‐incision antibiotics. At 5 years, 272 (9.0%) children had asthma, 672 (22.3%) had eczema and 180 (6.0%) had allergic rhinitis.

There were no substantial differences between those included in the analysis and those excluded for any reason (Figure 1), except women in the excluded group were younger (27.9 vs. 29.3 years), babies in the excluded group had lower birthweight (2950 vs. 3215 g) and children in the excluded group were less likely to have asthma (6.7% vs. 9.0%) and eczema (18.2% vs. 22.3%) at age 5.

The characteristics of the children and their mothers are shown in Table 1. Forty‐three percent (1308) of mothers were of Pakistani ethnicity, just over a third (1091) were of White British ethnicity and a fifth (614) belonged to other ethnic groups (Indian, White other, Black, Bangladeshi and mixed ethnicity). Mothers had a mean age of 29 years at delivery (range 15 to 46) with 31% (782) educated to degree level or equivalent. Fifty‐four percent (1546) of children were breastfed and two‐thirds (1990) had at least one antibiotic prescription before 2 years of age (Table 1). There were fewer women of White British ethnicity and a higher proportion of women of Pakistani ethnicity in the group exposed to pre‐incision antibiotics than in those unexposed (Table 1), and differences in characteristics which vary by ethnicity in Bradford [20]; women of Pakistani ethnicity are more likely to breastfeed, have more children (or live in households with extended family so higher numbers of children per household) and are less likely to smoke. Most of the exposed group were from BiBBS (Table S2).

**TABLE 1:** Cohort characteristics overall and by exposure to in utero prophylactic antibiotics. Values are frequency (%) or mean (SD).

	All	Unexposed	Exposed
	*N* = 3013 (100.0%)	*N* = 2434 (80.8%)	*N* = 579 (19.2%)
**Mother characteristics**			
Age at delivery	29.3 (SD 5.7)	29.0 (SD 5.7)	30.3 (SD 5.4)
Ethnicity			
White British	1091 (36.2%)	999 (41.0%)	92 (15.9%)
Pakistani	1308 (43.4%)	979 (40.2%)	329 (56.8%)
Other ethnicity	614 (20.4%)	456 (18.7%)	158 (27.3%)
Smoked during pregnancy			
No	2180 (85.6%)	1686 (84.6%)	494 (89.3%)
Yes	367 (14.3%)	308 (15.5%)	59 (10.7%)
Missing	466	440	26
Number of children aged < 16 years living in household			
None	927 (38.1%)	785 (39.3%)	142 (32.6%)
1	720 (29.6%)	602 (30.2%)	118 (27.1%)
2	474 (19.5%)	368 (18.4%)	106 (24.3%)
3+	312 (12.8%)	242 (12.2%)	70 (16.1%)
Missing	580	437	143
Educational attainment			
Less than degree level or equivalent	1735 (68.9%)	1386 (69.5%)	349 (66.6%)
Degree level or equivalent	782 (31.1%)	607 (30.5%)	175 (33.4%)
Missing	496	441	55
History of asthma	623 (20.7%)	523 (21.5%)	100 (17.3%)
History of eczema	600 (19.9%)	501 (20.6%)	99 (17.1%)
History of allergic rhinoconjunctivitis	676 (22.4%)	555 (22.8%)	121 (20.9%)
Antibiotics prescribed in first trimester	456 (15.1%)	365 (15.0%)	91 (15.7%)
Antibiotics prescribed in second trimester	466 (15.5%)	383 (15.7%)	83 (14.3%)
Antibiotics prescribed in third trimester	351 (11.7%)	294 (12.1%)	57 (9.8%)
Post‐operative infection in mother	102 (3.4%)	91 (3.7%)	11 (1.9%)
**Child characteristics**			
Sex			
Male	1595 (52.9%)	1302 (53.5%)	293 (50.6%)
Female	1418 (47.1%)	1132 (46.5%)	286 (49.4%)
Birthweight (grams)	3215 (663)	3227 (654)	3163 (696)
Missing	7	0	7
Ever breastfed			
No	1266 (45.8%)	1189 (50.4%)	137 (25.6%)
Yes	1546 (54.2%)	1172 (49.6%)	399 (74.4%)
Missing	116	73	43
Antibiotics prescribed before 2 years of age	1990 (66.1%)	1661 (68.2%)	329 (56.8%)
Asthma/wheeze diagnosis by 2 years	171 (5.7%)	140 (5.8%)	31 (5.4%)
Asthma diagnosis by 5 years	272 (9.0%)	225 (9.2%)	47 (8.1%)
Eczema diagnosis by 2 years	493 (16.4%)	417 (17.1%)	76 (13.1%)
Eczema diagnosis by 5 years	672 (22.3%)	572 (23.5%)	100 (17.3%)
Allergic rhinitis diagnosis by 2 years	82 (2.7%)	68 (2.8%)	14 (2.4%)
Allergic rhinitis by 5 years	180 (6.0%)	151 (6.2%)	29 (5.0%)

Unadjusted estimates showed that overall the risk of asthma, eczema and allergic rhinitis at age 5 and at age 2 was lower in children exposed to pre‐incision antibiotics than in children who were unexposed (Table 2). However, among boys, the risk of asthma/wheeze was slightly higher in the exposed than the unexposed group. Boys had a higher risk of asthma/wheeze than girls at age 5 and age 2, and the risk of eczema and allergic rhinitis was similar in boys and girls (Table 2 and Tables S3–S5).

**TABLE 2:** Diagnosis of asthma, eczema and allergic rhinitis by age 5 and 2, by exposure to in utero prophylactic antibiotics and sex.

	Diagnosis by age 5				Diagnosis by age 2			
	Boys		Girls		Boys		Girls	
	Unexposed *N* = 1302	Exposed *N* = 293	Unexposed *N* = 1132	Exposed *N* = 286	Unexposed *N* = 1302	Exposed *N* = 293	Unexposed *N* = 1132	Exposed *N* = 286
Asthma	134 (10.3%)	31 (10.6%)	91 (8.0%)	16 (5.6%)	90 (6.9%)	23 (7.9%)	50 (4.4%)	8 (2.8%)
Eczema	308 (23.7%)	53 (18.1%)	264 (23.3%)	47 (16.4%)	230 (17.7%)	39 (13.3%)	187 (16.5%)	37 (12.9%)
Allergic rhinitis	87 (6.7%)	15 (5.1%)	64 (5.7%)	14 (4.9%)	37 (2.8%)	6 (2.1%)	31 (2.7%)	8 (2.8%)

### 3.1 Outcomes at Age 5

**FIGURE 2:**
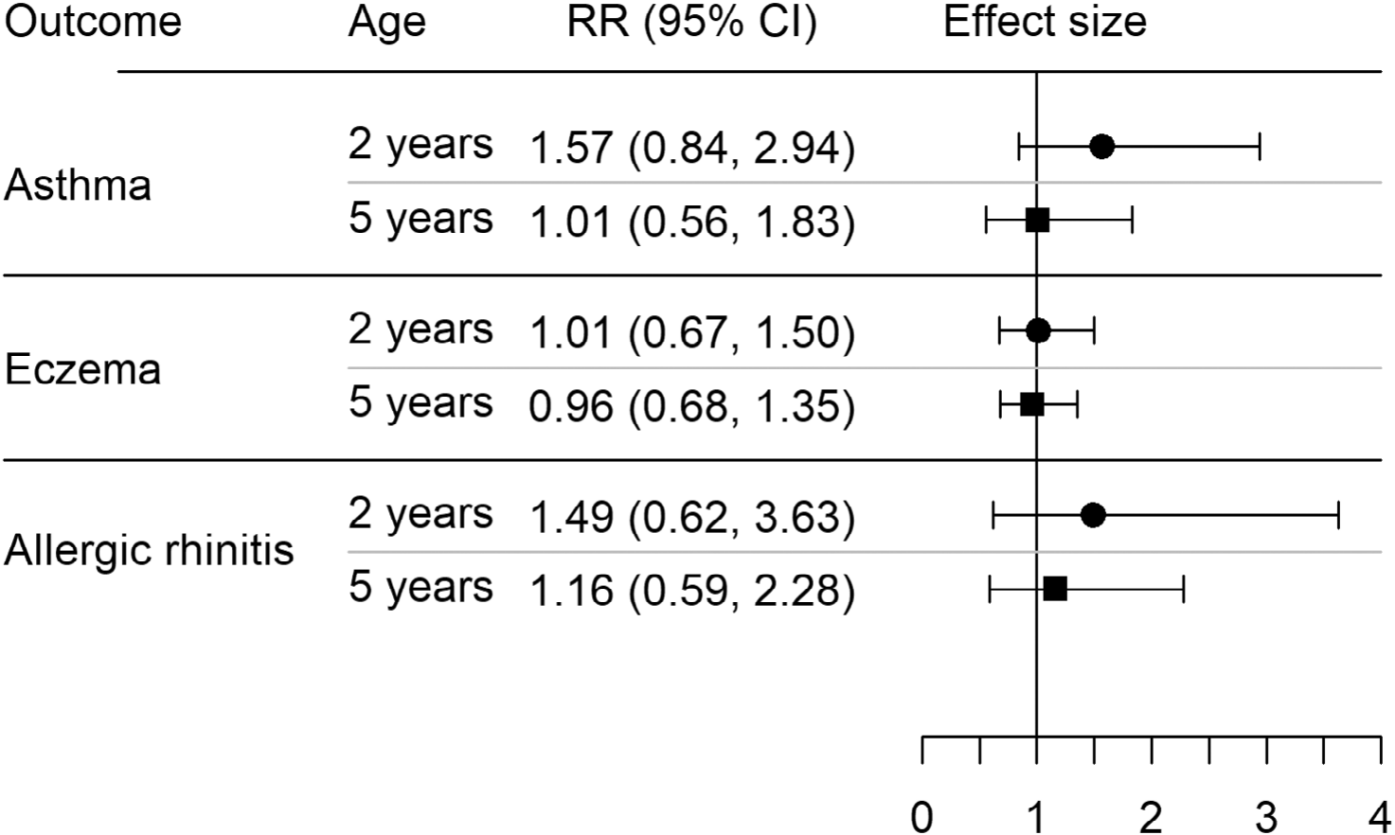
Adjusted risk ratios (RR) with 95% confidence intervals (95% CI) for the association between timing of intrapartum antibiotic prophylaxis administration and asthma, eczema and allergic rhinitis diagnosed by 2 and 5 years.

After adjustment for month/year of birth and other confounders, there was no evidence of an association between exposure to pre‐incision antibiotics (vs. antibiotics after cord clamping) and risk of asthma (adjusted risk ratio [aRR] 1.01, 95% CI 0.56, 1.83), eczema (aRR 0.96, 95% CI 0.68, 1.35) or allergic rhinitis (aRR 1.16, 95% CI 0.59, 2.28) at 5 years (Figure 2, Tables S3–S5). The corresponding adjusted absolute risks and adjusted risk differences (aRD) within 5 years (based on the rate difference) are: asthma 9.1% exposed, 9.0% unexposed, aRD 0.08 per 100 (95% CI −5.28, 5.44); eczema 21.5% exposed, 22.5% unexposed, aRD −1.01 per 100 (95% CI −8.65, 6.63); allergic rhinitis 6.8% exposed, 5.8% unexposed, aRD 0.88 per 100 (95% CI −3.17, 4.92).

### 3.2 Outcomes at Age 2

The adjusted risk ratio for wheeze at age 2 was higher than for asthma at age 5 (aRR 1.57, 95% CI 0.84, 2.94) but did not provide strong evidence of an increased risk for children exposed to pre‐incision antibiotics (Figure 2, Table S3). There was no evidence of an association between exposure to pre‐incision antibiotics (vs. antibiotics after cord clamping) and the risk of eczema (aRR 1.01, 95% CI 0.67, 1.50) or allergic rhinitis (aRR 1.49, 95% CI 0.62, 3.63) at age 2 (Figure 2, Tables S4 and S5, respectively).

Results of a complete case analysis indicated similar patterns (Tables S6–S8). Excluding children born from March 2018 onwards, who were 0–2 years during COVID‐19 restrictions (*n* = 199), did not substantially change the results (Table S9).

### 3.3 Maternal Surgical Site Infections (SSI) Following CS

Overall, 102 (3.4%) women had SSI following CS. A lower proportion of women who received pre‐incision antibiotics had infections than women receiving antibiotics after cord clamping: 11 (1.9%) versus 91 (3.7%) women (*X* ^2^ = 4.84, *p* = 0.028). The low number of infections precluded any further analysis.

## 4 Discussion

### 4.1 Main Findings

Our findings do not indicate an increased risk of asthma, eczema or allergic rhinitis at age 5 in children exposed to pre‐incision antibiotics during CS compared to those born by CS but not exposed to prophylactic antibiotics. The estimate for wheeze at 2 years (aRR 1.57, 95% CI 0.84, 2.94) could suggest a short‐term effect of pre‐incision antibiotic exposure, although the CI includes one and this finding must be interpreted with caution due to the non‐specific nature of wheeze in early childhood. The estimates for allergic rhinitis are imprecise, due to low numbers of children identified. We observed a lower proportion of maternal SSIs following CS in women who received prophylactic antibiotics pre‐incision compared to those receiving antibiotics after cord clamping.

### 4.2 Strengths and Limitations

The BiB study provided a unique opportunity for a natural experiment to investigate the long‐term impact of a change in the timing of prophylactic antibiotics for CS, as children enrolled in the BiB and BiBBS birth cohorts were born between 2007 and 2019, which includes the period when the timing of administration changed. Linkage to routine data was incorporated into the design of BiB cohorts from the start, so primary and secondary care records are available for nearly all children, and midwives, General Practitioners (GPs) and other healthcare professionals in Bradford have been trained in collecting and recording research‐quality routine data. Loss to follow‐up was minimal (1.3% in BiB and 1.8% in BiBBS) as outcome data are available from linked routine EHR without having to re‐contact participants. Our case definitions of asthma, eczema and allergic rhinitis were carefully developed to minimise misclassification [18]. Most children with asthma, wheeze or eczema would be seen by a GP and our incidence estimates are consistent with other UK studies [21, 22, 23]. Through review of paper maternity records for the BiB cohort, we determined the timing of antibiotic administration for each individual delivery. The wealth of study questionnaire data in BiB and BiBBS and linkage to primary and secondary care records enabled us to adjust for important confounders.

The change in practice to pre‐incision administration occurred over a longer period than expected based on our initial audit of maternity records, and we therefore expanded the study to include children from BiBBS. BiBBS includes families from three Bradford wards compared to all wards for BiB, which are more disadvantaged and diverse than Bradford overall. However, as the timing of antibiotic administration is not determined by individual maternal characteristics, this is unlikely to have introduced selection bias. We observed some differences in characteristics between the cohorts recruited at different time periods, particularly ethnicity, which likely explains differences in breastfeeding, family size and smoking. Some differences between the cohorts may be due to wider policy changes occurring over time, for example, antibiotic prescribing may have decreased due to greater prescriber awareness of antimicrobial resistance. There may have been a COVID‐19 effect, as some BiBBS children were < 2 years old during the pandemic and likely experienced fewer infections due to reduced contact with others during lockdowns, and were therefore less likely to be prescribed antibiotics [24]. We have adjusted for month/year of birth, in addition to ethnicity, maternal education (measure of socio‐economic position) and other confounders, to account for differences between cohorts and time trends in risk factors for, and diagnosis of, asthma, eczema and allergic rhinitis. There may be some residual confounding due to the non‐randomised comparison. Paternal history of asthma, eczema and allergic rhinitis was not available for most children so could not be included as a confounder.

Classification of the timing of antibiotics administration from paper records was more complex than expected, so exposure misclassification is possible. However, we developed detailed rules to mitigate this. Any misclassification would have been unrelated to the outcomes, as the researcher extracting the data was unaware of the outcome status of each child. In some cases, the maternity notes could not be found or the timing of antibiotics was unclear and these children were excluded from the final sample. There were few differences between those included in the analysis and those excluded for any reason. BiBBS children were categorised as exposed to pre‐incision antibiotics based on the policy in place and our checks of a random sample of maternity records, but we acknowledge that exposure misclassification is possible, especially for children born by emergency CS whose mothers may have received antibiotics late during the procedure. Data on the type of CS (elective or emergency) for the BiBBS cohort are not yet available for analysis, but NHS Digital data for BRI for 2016–2019 indicate that the proportion of emergency CS (vs. elective CS) was lower than that observed for the BiB cohort (2007–2011). Similar proportions of women of Pakistani ethnicity and white British ethnicity had an emergency CS, based on BiB data. We therefore do not expect that emergency CS in the BiBBS cohort would have substantially affected exposure classification.

Diagnosis of allergic rhinitis is likely to be under‐reported in primary care records as parents may only consult a pharmacist for allergic rhinitis symptoms and over‐the‐counter medications. The smaller number of cases led to imprecise estimates, and potential detection biases; children with more GP visits for any reason could have a higher probability of being diagnosed with allergic rhinitis. Intrapartum antibiotics are administered to prevent neonatal infection if the mother has group B streptococcal (GBS) colonisation, bacteriuria or infection [25]. These are not given for elective CS but may be given to women delivering by emergency CS who have been in labour. In our study, only 4.4% of women in BiB had antibiotics during labour (65 cefuroxime and metronidazole, 45 other antibiotics). For BiBBS it was not possible to identify antibiotics given specifically during labour from pharmacy records, but similarly low numbers are likely, especially as the proportion of emergency CS deliveries was likely lower in BiBBS than BiB. This extra antibiotic exposure was therefore unlikely to have affected our results. Our analysis of maternal SSI was based on routine data (not active surveillance) and we were unable to adjust for key clinical risk factors such as emergency versus elective CS, maternal BMI or diabetes. We could not accurately identify cases of endometritis, cystitis or sepsis clearly distinguished from a general SSI code. Our study was in a single centre with all women delivering at BRI, but as a multi‐ethnic urban cohort our findings are likely to be generalisable to many populations in the UK and Europe.

### 4.3 Interpretation (In Light of Other Evidence)

The key strength of our study is individual‐level data on timing of antibiotic administration. This contrasts with a large national UK study where the timing of antibiotic administration was estimated through a survey of clinical directors for maternity care in all hospitals performing CS [26]. Šumilo et al. used UK EHR for primary care and Hospital Episode Statistics data for England and found no evidence of an association between pre‐incision antibiotics for CS and the risk of asthma (incidence rate ratio, IRR 0.91, 95% CI 0.78, 1.05) or eczema (IRR 0.98, 95% CI 0.94, 1.03) at age 5 [26, 27]. The consistency of our results, despite different study designs and potential biases, increases the reliability of our findings.

Our findings are also consistent with a large US retrospective cohort study which found little evidence of an increased risk of asthma or allergic rhinitis in children exposed to intrapartum antibiotics (for CS or for GBS prevention in vaginal deliveries). Timing of antibiotics administration for CS was not investigated; exposure to intrapartum antibiotics was defined as any intravenous antibiotic given during the delivery admission prior to the time of delivery [28].

Exposure to antibiotics early in life could affect the risk of asthma and allergic disease via an impact on the gut microbiome. Studies in which the gut microbiota were profiled in neonates and infants have demonstrated disruption to gut colonization in babies born by CS, in those exposed to antibiotics or not breastfed among babies born vaginally [29], and in babies born by both CS and vaginal delivery whose mothers received intrapartum antibiotics [30]. Dysbiosis during this critical window of immune development can affect immune responses and tolerance, and may predispose to Th‐2 skewed or allergic responses [11, 12, 13].

Our findings on exposure to prophylactic antibiotics, and those of Šumilo et al. [26], contrast with studies investigating the effect of prenatal (through maternal antibiotic use in pregnancy) and early life exposure to antibiotics on asthma, which include all children regardless of mode of birth. In our analysis of the whole BiB cohort, antibiotic prescriptions to the mother in late pregnancy were associated with an increased odds of asthma at 5–8 years (adjusted OR 1.40, 95% CI 1.05, 1.87), as were prescriptions to the child in the first 2 years (adjusted OR 2.00, 95% CI 1.71, 2.34) [3]. Children born by CS have altered gut microbiota related to mode of birth [29], so the additional impact of a single dose of antibiotics just before birth may be minimal. We observed larger effect sizes among children born by CS (prenatal: 1.68 [1.13; 2.51], postnatal: 2.19 [1.63; 2.93]), compared to vaginal delivery (prenatal: 1.31 [0.99; 1.75], postnatal: 2.01 [1.71; 2.36]), indicating a possible additive effect with mode of birth [3]. However, findings from the Danish National Birth Cohort showed an association between antibiotics in pregnancy and asthma at 11 years only among children born by vaginal delivery and not in those born by CS [31]. Prophylactic antibiotics for CS are broad spectrum, usually cefuroxime and metronidazole. Antibiotics prescribed during pregnancy and in early childhood may be more likely to target specific infections, and different types of antibiotics may have different effects on the gut microbiome, which could translate to different impacts on risk of asthma, eczema and allergic rhinitis [32].

## 5 Conclusion

This study contributes to the evidence base informing the optimum time to administer prophylactic antibiotics to women for CS, balancing the risks and benefits to the mother and her child. The available combined evidence is reassuring for women and their families, and supports the current UK and international guidelines which recommend pre‐incision antibiotics to reduce the risk of maternal infections. However, our findings cannot rule out an increased risk of asthma, eczema and allergic rhinitis in children exposed to pre‐incision antibiotics. It will be important to continue to monitor the longer‐term impacts of prophylactic antibiotic exposure into adolescence, using EHR combined with research data from birth cohorts to have greater confidence in the longer‐term safety of these policy changes.

## Author Contributions

L.P., G.S., S.O., E.S.P., N.P. and J.W. conceived and designed the study. S.S.C. compiled the data, developed the outcome definitions, derived the variables, developed the statistical analysis plan and conducted preliminary analysis, with G.S. and L.P. A.H. compiled the BiBBS data. G.S. conducted the statistical analysis, and L.P. drafted and revised the manuscript. All authors interpreted the results, revised the manuscript and approved the final version.

## Ethics Statement

This study has Health Research Authority (HRA) and Health and Care Research Wales (HCRW) approval (ref: 238908). The Born in Bradford project has ethical approval from the Bradford Research Ethics Committee (ref: 07/H1302/112).

## Consent

Women recruited to BiB and BiBBS gave informed consent for their participation and on behalf of their children.

## Conflicts of Interest

The authors declare no conflicts of interest.

## Supporting information


**Table S1:** Read/ICD‐10 codes used to identify surgical site infection.
**Table S2:** Cohort characteristics in BiB and BiBBS. Values are frequency (%) or mean (SD).
**Table S3:** Risk ratios (RR) with 95% confidence intervals (95% CI) for the association between timing of intrapartum antibiotic prophylaxis administration and asthma diagnosed between 3 and 5 years, and wheeze by 2 years. Analyses use the imputed data.
**Table S4:** Risk ratios (RR) with 95% confidence intervals (95% CI) for the association between timing of intrapartum antibiotic prophylaxis administration and eczema diagnosed by 5 and 2 years. Analyses use the imputed data.
**Table S5:** Risk ratios (RR) with 95% confidence intervals (95% CI) for the association between timing of intrapartum antibiotic prophylaxis administration and allergic rhinitis diagnosed by 5 and 2 years. Analyses use the imputed data.
**Table S6:** Risk ratios (RR) with 95% confidence intervals (95% CI) for the association between timing of intrapartum antibiotic prophylaxis administration and asthma diagnosed between 3 and 5 years, and wheeze by 2 years. Complete case analysis.
**Table S7:** Risk ratios (RR) with 95% confidence intervals (95% CI) for the association between timing of intrapartum antibiotic prophylaxis administration and eczema diagnosed between by 5 and 2 years. Complete case analysis.
**Table S8:** Risk ratios (IRR) with 95% confidence intervals (95% CI) for the association between timing of intrapartum antibiotic prophylaxis administration and allergic rhinitis diagnosed between by 5 and 2 years. Complete case analysis.
**Table S9:** Results of sensitivity analysis excluding children born March 2018 onwards who were 0–2 years during COVID‐19 restrictions.
**Figure S1:** Directed Acyclic Graph for effect of timing of prophylactic antibiotics for caesarean section (pre‐incision vs. post cord clamping) on risk of asthma, eczema and allergic rhinitis at age 5.

## Data Availability

Born in Bradford encourages data requests from researchers and follows the FAIR principles. The BiB data dictionary can be found here: https://borninbradford.github.io/datadict/ and requests made here: https://borninbradford.nhs.uk/our‐data/how‐to‐access‐data/.
