# Supplementary material for: Timing of Intrapartum Antibiotics at Caesarean Section and Risk of Asthma, Eczema and Allergic Rhinitis: Results From a Natural Experiment
Source: BJOG. 2025 Nov 14;133(4):698–707. doi: 10.1111/1471-0528.70083 (PMC12884210; doi:10.1111/1471-0528.70083)
Supplement: Supplementary file 1 — Table S1: Read/ICD‐10 codes used to identify surgical site infection. Table S2: Cohort characteristics in BiB and BiBBS. Values are frequency (%) or mean (SD). Table S3: Risk ratios (RR) with 95% confidence intervals (95% CI) for the association between timing of intrapartum antibiotic prophylaxis administration and asthma diagnosed between 3 and 5 years, and wheeze by 2 years. Analyses use the imputed data. Table S4: Risk ratios (RR) with 95% confidence intervals (95% CI) for the association between timing of intrapartum antibiotic prophylaxis administration and eczema diagnosed by 5 and 2 years. Analyses use the imputed data. Table S5: Risk ratios (RR) with 95% confidence intervals (95% CI) for the association between timing of intrapartum antibiotic prophylaxis administration and allergic rhinitis diagnosed by 5 and 2 years. Analyses use the imputed data. Table S6: Risk ratios (RR) with 95% confidence intervals (95% CI) for the association between timing of intrapartum antibiotic prophylaxis administration and asthma diagnosed between 3 and 5 years, and wheeze by 2 years. Complete case analysis. Table S7: Risk ratios (RR) with 95% confidence intervals (95% CI) for the association between timing of intrapartum antibiotic prophylaxis administration and eczema diagnosed between by 5 and 2 years. Complete case analysis. Table S8: Risk ratios (IRR) with 95% confidence intervals (95% CI) for the association between timing of intrapartum antibiotic prophylaxis administration and allergic rhinitis diagnosed between by 5 and 2 years. Complete case analysis. Table S9: Results of sensitivity analysis excluding children born March 2018 onwards who were 0–2 years during COVID‐19 restrictions. Figure S1: Directed Acyclic Graph for effect of timing of prophylactic antibiotics for caesarean section (pre‐incision vs. post cord clamping) on risk of asthma, eczema and allergic rhinitis at age 5. [file BJO-133-698-s001.docx]

**Supplementary figure and tables**

Figure S1:

Directed Acyclic Graph for effect of timing of prophylactic antibiotics for caesarean section (pre-incision vs post cord clamping) on risk of asthma, eczema and allergic rhinitis at age 5.


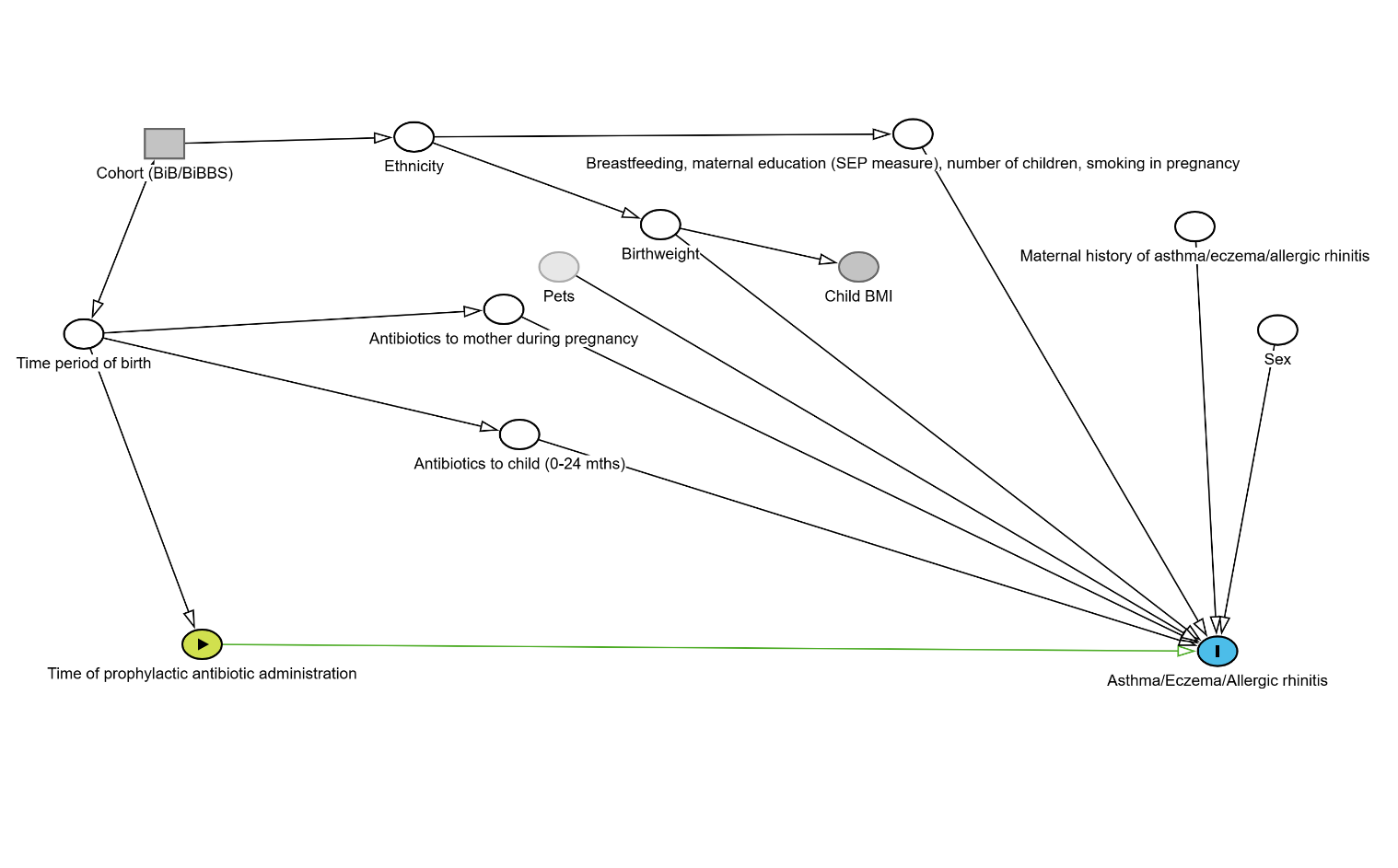


Table S1:

Read / ICD-10 codes used to identify surgical site infection

| **Read code** | **Description** |
| --- | --- |
| Xa6nk | Postoperative wound infection |
| SP255 | Postoperative wound infection, unspecified |
| SP257 | Postoperative wound infection-superficial |
| L3945 | Infection of obstetric surgical wound |
| SP25. | Postoperative infection |
| XaBMa | MRSA infection of postoperative wound |
| XaB1t | Postoperative wound cellulitis |
| SP250 | Postoperative stitch abscess |
| SP256 | Postoperative wound infection-deep |

Table S2: Cohort characteristics in BiB and BiBBS. Values are frequency (%) or mean (SD)

|  | **All** | **BiB cohort** | | **BiBBS cohort** |
| --- | --- | --- | --- | --- |
|  |  | **Unexposed** | **Exposed** | **Exposed** |
|  | N=3,013 (100.0%) | N=2,434  (80.8%) | N=94  (3.1%) | N=485  (16.1%) |
| **Mother characteristics** |  |  |  |  |
| Age at delivery | 29.3 (SD 5.7) | 29.0 (SD 5.7) | 29.2 (SD 5.3) | 30.5 (SD 5.4) |
| Ethnicity |  |  |  |  |
| White British | 1,091 (36.2%) | 999 (41.0%) | 37 (39.4%) | 55 (11.3%) |
| Pakistani | 1,308 (43.4%) | 979 (40.2%) | 36 (38.3%) | 293 (60.4%) |
| Other ethnicity | 614 (20.4%) | 456 (18.7%) | 21 (22.3%) | 137 (28.3%) |
| Smoked during pregnancy |  |  |  |  |
| No | 2,180 (85.6%) | 1,686 (84.6%) | 69 (87.3%) | 425 (89.7%) |
| Yes | 367 (14.3%) | 308 (15.5%) | 10 (12.7%) | 49 (10.3%) |
| Missing | 466 | 437 | 15 | 11 |
| Number of children aged <16 years living in household |  |  |  |  |
| None | 927 (38.1%) | 785 (39.3%) | 38 (48.1%) | 104 (29.1%) |
| 1 | 720 (29.6%) | 602 (30.2%) | 25 (31.7%) | 93 (26.1%) |
| 2 | 474 (19.5%) | 368 (18.4%) | 9 (11.4%) | 97 (27.2%) |
| 3+ | 312 (12.8%) | 242 (12.2%) | 7 (8.9%) | 63 (17.7%) |
| Missing | 580 | 441 | 15 | 128 |
| Educational attainment |  |  |  |  |
| Less than degree level or equivalent | 1,735 (68.9%) | 1,386 (69.5%) | 48 (61.5%) | 301 (67.5%) |
| Degree level or equivalent | 782 (31.1%) | 607 (30.5%) | 30 (38.5%) | 145 (32.5%) |
| Missing | 496 | 440 | 16 | 39 |
| History of asthma | 623 (20.7%) | 523 (21.5%) | 23 (24.5%) | 77 (15.9%) |
| History of eczema | 600 (19.9%) | 501 (20.6%) | 19 (20.2%) | 80 (16.5%) |
| History of allergic rhinoconjunctivitis | 676 (22.4%) | 555 (22.8%) | 24 (25.5%) | 97 (20.0%) |
| Antibiotics prescribed in first trimester | 458 (15.2%) | 365 (15.0%) | 6 (6.4%) | 87 (17.9%) |
| Antibiotics prescribed in second trimester | 466 (15.5%) | 383 (15.7%) | 18 (19.2%) | 65 (13.4%) |
| Antibiotics prescribed in third trimester | 351 (11.7%) | 294 (12.1%) | 19 (20.2%) | 38 (7.8%) |
| Post-operative infection in mother | 102 (3.4%) | 91 (3.7%) | 1 (1.1%) | 10 (2.1%) |
| **Child characteristics** |  |  |  |  |
| Sex |  |  |  |  |
| Male | 1,595 (52.9%) | 1,302 (53.5%) | 48 (51.1%) | 245 (50.5%) |
| Female | 1,418 (47.1%) | 1,132 (46.5%) | 46 (48.9%) | 240 (49.5%) |
| Birthweight (grams) | 3215 (663) | 3227 (654) | 3290 (633) | 3138 (706) |
| Missing | 7 | 0 | 7 | 0 |
| Ever breastfed |  |  |  |  |
| No | 1,266 (45.8%) | 1,189 (50.4%) | 45 (50.0%) | 90 (20.6%) |
| Yes | 1,546 (54.2%) | 1,172 (49.6%) | 45 (50.0%) | 356 (79.4%) |
| Missing | 111 | 73 | 4 | 39 |
| Antibiotics prescribed before 2 years of age | 1,990 (66.1%) | 1,611 (68.2%) | 61 (64.9%) | 268 (55.3%) |
| Asthma/wheeze diagnosis by 2 years | 171 (5.7%) | 140 (5.8%) | 8 (8.5%) | 23 (4.7%) |
| Asthma diagnosis by 5 years | 272 (9.0%) | 225 (9.2%) | 10 (10.6%) | 37 (7.6%) |
| Eczema diagnosis by 2 years | 493 (16.4%) | 417 (17.1%) | 16 (17.0%) | 60 (12.4%) |
| Eczema diagnosis by 5 years | 672 (22.3%) | 572 (23.5%) | 22 (23.4%) | 78 (16.1%) |
| Allergic rhinitis diagnosis by 2 years | 82 (2.7%) | 68 (2.8%) | 3 (3.2%) | 11 (2.3%) |
| Allergic rhinitis by 5 years | 180 (6.0%) | 151 (6.2%) | 7 (7.5%) | 22 (4.5%) |

Table S3: Risk ratios (RR) with 95% confidence intervals (95% CI) for the association between timing of intrapartum antibiotic prophylaxis administration and asthma diagnosed between 3 and 5 years, and wheeze by 2 years. Analyses use the imputed data.

|  | Asthma diagnosed between 3- 5 years | | Wheeze diagnosed by 2 years | | |
| --- | --- | --- | --- | --- | --- |
|  | Unadjusted | Adjusted | Unadjusted | Adjusted |  |
| Prophylactic antibiotics administered before CS | 0.88 (0.65, 1.19) | - 1. (0.56, 1.83) | 0.93 (0.64, 1.36) | 1.57 (0.84, 2.94) |  |
| Ethnicity |  |  |  |  |  |
| White British | 1.00 | 1.00 | 1.00 | 1.00 |  |
| Pakistani | 1.38 (1.07, 1.77) | 1.40 (1.05, 1.85) | 1.34 (0.97, 1.85) | 1.24 (0.86, 1.77) |  |
| Other ethnicity | 0.72 (0.49, 1.04) | 0.84 (0.56, 1.24) | 0.79 (0.50, 1.26) | 0.89 (0.56, 1.41) |  |
| Smoked during pregnancy | 0.92 (0.62, 1.35) | 0.93 (0.62, 1.41) | 0.84 (0.52, 1.35) | 0.81 (0.49, 1.33) |  |
| Number of children aged <16 years living in household |  |  |  |  |  |
| None | 1.00 | 1.00 | 1.00 | 1.00 |  |
| 1 | 1.02 (0.74, 1.40) | 0.96 (0.70, 1.33) | 1.11 (0.75, 1.65) | 1.07 (0.72, 1.58) |  |
| 2 | 1.14 (0.82, 1.58) | 1.01 (0.73, 1.41) | 1.42 (0.92, 2.18) | 1.30 (0.84, 1.99) |  |
| 3+ | 1.26 (0.86, 1.84) | 1.06 (0.71, 1.58) | 1.53 (0.96, 2.43) | 1.34 (0.82, 2.19) |  |
| Degree level educational attainment | 0.94 (0.71, 1.25) | 1.01 (0.76, 1.35) | 0.83 (0.59, 1.18) | 0.88 (0.62, 1.26) |  |
| Mother history of asthma | 2.13 (1.69, 2.68) | 2.05 (1.63, 2.59) | 2.13 (1.57, 2.88) | 2.07 (1.52, 2.81) |  |
| Antibiotics prescribed in first trimester | 1.26 (0.94, 1.69) | 1.02 (0.76, 1.37) | 1.39 (0.97, 2.00) | 1.16 (0.80, 1.68) |  |
| Antibiotics prescribed in second trimester | 1.29 (0.97, 1.72) | 1.03 (0.77, 1.38) | 1.31 (0.91, 1.89) | 1.00 (0.68, 1.48) |  |
| Antibiotics prescribed in third trimester | 1.27 (0.92, 1.75) | 1.03 (0.75, 1.41) | 1.36 (0.91, 2.03) | 1.11 (0.73, 1.66) |  |
| Female sex | 0.73 (0.58, 0.92) | 0.76 (0.60, 0.96) | 0.58 (0.42, 0.79) | 0.58 (0.42, 0.79) |  |
| Birthweight (grams) | 1.00 (1.00, 1.00) | 1.00 (1.00, 1.00) | 1.00 (1.00, 1.00) | 1.00 (1.00, 1.00) |  |
| Child ever breastfed | 0.88 (0.70, 1.10) | 0.96 (0.76, 1.22) | 0.93 (0.69, 1.25) | 1.06 (0.78, 1.44) |  |
| Antibiotics prescribed to child before 2 years of age | 2.98 (2.14, 4.13) | 2.68 (1.93, 3.73) | 2.42 (1.64, 3.56) | 2.07 (1.41, 3.06) |  |
| Month/year of birth | 1.00 (1.00, 1.00) | 1.00 (0.99, 1.01) | 1.00 (0.99, 1.00) | 0.99 (0.99, 1.00) |  |

Table S4: Risk ratios (RR) with 95% confidence intervals (95% CI) for the association between timing of intrapartum antibiotic prophylaxis administration and eczema diagnosed by 5 and 2 years. Analyses use the imputed data.

|  | Eczema diagnosed by 5 years | | | Eczema diagnosed by 2 years | | |
| --- | --- | --- | --- | --- | --- | --- |
|  | Unadjusted | Adjusted | Unadjusted | | Adjusted |  |
| Prophylactic antibiotics administered before CS | 0.73 (0.61, 0.89) | 0.96 (0.68, 1.35) | 0.77 (0.61, 0.96) | | 1.01 (0.67, 1.50) |  |
| Ethnicity |  |  |  | |  |  |
| White British | 1.00 | 1.00 | 1.00 | | 1.00 |  |
| Pakistani | 1.24 (1.06, 1.44) | 1.30 (1.10, 1.54) | 1.26 (1.04, 1.51) | | 1.34 (1.09, 1.65) |  |
| Other ethnicity | 1.22 (1.02, 1.48) | 1.36 (1.12, 1.66) | 1.16 (0.92, 1.46) | | 1.28 (1.01, 1.62) |  |
| Smoked during pregnancy | 0.94 (0.76, 1.15) | 1.00 (0.80, 1.25) | 0.90 (0.69, 1.17) | | 1.00 (0.76, 1.32) |  |
| Number of children aged <16 years living in household |  |  |  | |  |  |
| None | 1.00 | 1.00 | 1.00 | | 1.00 |  |
| 1 | 0.97 (0.82, 1.16) | 0.93 (0.78, 1.10) | 0.92 (0.75, 1.13) | | 0.87 (0.71, 1.07) |  |
| 2 | 0.76 (0.61, 0.95) | 0.72 (0.58, 0.90) | 0.72 (0.55, 0.94) | | 0.67 (0.52, 0.88) |  |
| 3+ | 0.89 (0.70, 1.13) | 0.80 (0.62, 1.02) | 0.89 (0.66, 1.18) | | 0.80 (0.59, 1.08) |  |
| Degree level educational attainment | 1.02 (0.87, 1.20) | 0.98 (0.83, 1.16) | 1.06 (0.88, 1.28) | | 1.01 (0.83, 1.23) |  |
| Mother history of eczema | 1.61 (1.40, 1.85) | 1.50 (1.30, 1.72) | 1.49 (1.24, 1.78) | | 1.39 (1.15, 1.65) |  |
| Antibiotics prescribed in first trimester | 1.26 (1.06, 1.49) | 1.19 (1.00, 1.41) | 1.14 (0.92, 1.41) | | 1.08 (0.87, 1.35) |  |
| Antibiotics prescribed in second trimester | 1.18 (1.00, 1.41) | 1.07 (0.90, 1.27) | 1.14 (0.92, 1.41) | | 1.06 (0.85, 1.31) |  |
| Antibiotics prescribed in third trimester | 1.04 (0.85, 1.27) | 0.92 (0.75, 1.14) | 0.93 (0.72, 1.21) | | 0.84 (0.64, 1.09) |  |
| Female sex of child | 0.97 (0.85, 1.11) | 1.00 (0.88, 1.15) | 0.94 (0.80, 1.10) | | 0.98 (0.84, 1.16) |  |
| Birthweight (grams) | 1.00 (1.00, 1.00) | 1.00 (1.00, 1.00) | 1.00 (1.00, 1.00) | | 1.00 (1.00, 1.00) |  |
| Child ever breastfed | 0.97 (0.85, 1.11) | 1.01 (0.88, 1.16) | 1.03 (0.87, 1.21) | | 1.05 (0.89, 1.24) |  |
| Antibiotics prescribed to child before 2 years of age | 1.77 (1.50, 2.09) | 1.66 (1.40, 1.96) | 1.97 (1.61, 2.42) | | 1.89 (1.53, 2.33) |  |
| Month/year of birth | 1.00 (0.99, 1.00) | 1.00 (0.99, 1.00) | 1.00 (0.99, 1.00) | | 1.00 (0.99, 1.00) |  |

Table S5: Risk ratios (RR) with 95% confidence intervals (95% CI) for the association between timing of intrapartum antibiotic prophylaxis administration and allergic rhinitis diagnosed by 5 and 2 years. Analyses use the imputed data.

|  | Allergic rhinitis diagnosed by 5 years | | Allergic rhinitis diagnosed by 2 years | |
| --- | --- | --- | --- | --- |
|  | Unadjusted | Adjusted | Unadjusted | Adjusted |
| Prophylactic antibiotics administered before CS | 0.81 (0.55, 1.19) | 1.16 (0.59, 2.28) | 0.87 (0.49, 1.53) | 1.49 (0.62, 3.63) |
| Ethnicity |  |  |  |  |
| White British | 1.00 | 1.00 | 1.00 | 1.00 |
| Pakistani | 1.90 (1.34, 2.70) | 1.97 (1.35, 2.89) | 1.24 (0.76, 2.00) | 1.29 (0.76, 2.19) |
| Other ethnicity | 1.61 (1.06, 2.46) | 1.75 (1.15, 2.67) | 0.99 (0.53, 1.84) | 1.14 (0.61, 2.12) |
| Smoked during pregnancy | 0.73 (0.43, 1.21) | 0.90 (0.53, 1.55) | 0.89 (0.45, 1.75) | 0.87 (0.41, 1.82) |
| Number of children aged <16 years living in household |  |  |  |  |
| None | 1.00 | 1.00 | 1.00 | 1.00 |
| 1 | 1.15 (0.81, 1.64) | 1.06 (0.74, 1.53) | 1.08 (0.64, 1.84) | 1.01 (0.59, 1.72) |
| 2 | 0.86 (0.54, 1.36) | 0.72 (0.45, 1.16) | 0.87 (0.44, 1.71) | 0.76 (0.38, 1.54) |
| 3+ | 0.73 (0.40, 1.34) | 0.55 (0.30, 1.03) | 0.59 (0.23, 1.49) | 0.47 (0.18, 1.21) |
| Degree level educational attainment | 1.00 (0.72, 1.38) | 0.87 (0.62, 1.21) | 0.89 (0.54, 1.48) | 0.85 (0.51, 1.43) |
| Mother history of hay fever | 2.47 (1.86, 3.28) | 2.29 (1.72, 3.07) | 2.45 (1.59, 3.77) | 2.31 (1.48, 3.61) |
| Antibiotics prescribed in first trimester | 1.12 (0.77, 1.64) | 1.03 (0.71, 1.51) | 1.58 (0.94, 2.64) | 1.42 (0.85, 2.38) |
| Antibiotics prescribed in second trimester | 1.18 (0.82, 1.71) | 1.02 (0.70, 1.47) | 1.33 (0.77, 2.27) | 1.06 (0.62, 1.80) |
| Antibiotics prescribed in third trimester | 1.22 (0.81, 1.84) | 1.07 (0.70, 1.63) | 1.70 (0.98, 2.94) | 1.40 (0.78, 2.51) |
| Female sex of child | 0.86 (0.65, 1.15) | 0.89 (0.67, 1.18) | 1.02 (0.66, 1.57) | 1.05 (0.69, 1.60) |
| Birthweight (grams) | 1.00 (1.00, 1.00) | 1.00 (1.00, 1.00) | 1.00 (1.00, 1.00) | 1.00 (1.00, 1.00) |
| Child ever breastfed | 1.01 (0.76, 1.35) | 1.03 (0.77, 1.38) | 0.75 (0.49, 1.16) | 0.78 (0.51, 1.18) |
| Antibiotics prescribed to child before 2 years of age | 1.54 (1.11, 2.14) | 1.36 (0.97, 1.89) | 1.31 (0.82, 2.12) | 1.11 (0.68, 1.82) |
| Month/year of birth | 1.00 (0.99, 1.00) | 0.99 (0.99, 1.00) | 1.00 (0.99, 1.00) | 0.99 (0.98, 1.00) |

Table S6: Risk ratios (RR) with 95% confidence intervals (95% CI) for the association between timing of intrapartum antibiotic prophylaxis administration and asthma diagnosed between 3 and 5 years, and wheeze by 2 years. Complete case analysis.

|  | Asthma diagnosed between 3 and 5 years | | Wheeze diagnosed by 2 years | |
| --- | --- | --- | --- | --- |
|  | Unadjusted | Adjusted | Unadjusted | Adjusted |
| Prophylactic antibiotics administered before CS | 0.88 (0.65, 1.19) | 1.20 (0.66, 2.20) | 0.93 (0.64, 1.36) | 1.34 (0.66, 2.72) |
| Ethnicity |  |  |  |  |
| White British | 1.00 | 1.00 | 1.00 | 1.00 |
| Pakistani | 1.38 (1.07, 1.77) | 1.18 (0.87, 1.61) | 1.34 (0.96, 1.85) | 1.20 (0.82, 1.77) |
| Other ethnicity | 0.72 (0.49, 1.04) | 0.86 (0.56, 1.32) | 0.79 (0.47, 1.26) | 0.93 (0.56, 1.53) |
| Smoked during pregnancy | 0.94 (0.66, 1.36) | 0.86 (0.58, 1.28) | 0.77 (0.47, 1.26) | 0.74 (0.44, 1.24) |
| Number of children aged <16 years living in household |  |  |  |  |
| None | 1.00 | 1.00 | 1.00 | 1.00 |
| 1 | 1.02 (0.74, 1.40) | 0.99 (0.72, 1.36) | 1.12 (0.74, 1.69) | 1.05 (0.70, 1.58) |
| 2 | 1.15 (0.82, 1.63) | 1.09 (0.77, 1.54) | 1.49 (0.97, 2.29) | 1.30 (0.85, 2.01) |
| 3+ | 1.33 (0.91, 1.94) | 1.16 (0.78, 1.74) | 1.68 (1.06, 2.67) | 1.43 (0.87, 2.37) |
| Degree level educational attainment | 0.92 (0.70, 1.21) | 1.02 (0.77, 1.36) | 0.79 (0.56, 1.13) | 0.84 (0.59, 1.19) |
| Mother history of asthma | 2.13 (1.69, 2.68) | 1.99 (1.54, 2.57) | 2.13 (1.57, 2.88) | 2.10 (1.52, 2.91) |
| Antibiotics prescribed in first trimester | 1.26 (0.94, 1.69) | 0.97 (0.69, 1.37) | 1.39 (0.97, 2.00) | 0.98 (0.63, 1.49) |
| Antibiotics prescribed in second trimester | 1.29 (0.97, 1.72) | 1.07 (0.77, 1.49) | 1.31 (0.91, 1.89) | 1.08 (0.71, 1.65) |
| Antibiotics prescribed in third trimester | 1.27 (0.92, 1.75) | 0.93 (0.64, 1.34) | 1.36 (0.90, 2.03) | 0.95 (0.59, 1.53) |
| Female sex of child | 0.73 (0.58, 0.92) | 0.67 (0.52, 0.88) | 0.58 (0.42, 0.79) | 0.56 (0.40, 0.78) |
| Birthweight (grams) | 1.00 (1.00, 1.00) | 1.00 (1.00, 1.00) | 1.00 (1.00, 1.00) | 1.00 (1.00, 1.00) |
| Child ever breastfed | 0.88 (0.70, 1.11) | 0.95 (0.73, 1.24) | 0.93 (0.70, 1.25) | 1.11 (0.80, 1.55) |
| Antibiotics prescribed to child before 2 years of age | 2.98 (2.15, 4.13) | 2.54 (1.76, 3.66) | 2.42 (1.64, 3.56) | 2.05 (1.35, 3.13) |
| Month/year of birth | 1.00 (0.99, 1.01) | 1.00 (0.99, 1.01) | 1.00 (0.99, 1.01) | 1.00 (0.99, 1.01) |

Table S7: Risk ratios (RR) with 95% confidence intervals (95% CI) for the association between timing of intrapartum antibiotic prophylaxis administration and eczema diagnosed between by 5 and 2 years. Complete case analysis.

|  | Eczema diagnosed by 5 years | | | Eczema diagnosed by 2 years | | |
| --- | --- | --- | --- | --- | --- | --- |
|  | Unadjusted | Adjusted | Unadjusted | | Adjusted |  |
| Prophylactic antibiotics administered before CS | 0.73 (0.61, 0.89) | 1.03 (0.72, 1.47) | 0.77 (0.61, 0.96) | | 1.08 (0.70, 1.65) |  |
| Ethnicity |  |  |  | |  |  |
| White British | 1.00 | 1.00 | 1.00 | | 1.00 |  |
| Pakistani | 1.24 (1.06, 1.44) | 1.29 (1.07, 1.55) | 1.26 (1.04, 1.51) | | 1.29 (1.03, 1.61) |  |
| Other ethnicity | 1.22 (1.02, 1.48) | 1.33 (1.07, 1.65) | 1.16 (0.92, 1.46) | | 1.26 (0.97, 1.64) |  |
| Smoked during pregnancy | 0.96 (0.78, 1.18) | 1.00 (0.80, 1.25) | 0.91 (0.71, 1.18) | | 0.97 (0.74, 1.28) |  |
| Number of children aged <16 years living in household |  |  |  | |  |  |
| None | 1.00 | 1.00 | 1.00 | | 1.00 |  |
| 1 | 0.99 (0.82, 1.16) | 0.91 (0.77, 1.08) | 0.91 (0.74, 1.13) | | 0.85 (0.69, 1.05) |  |
| 2 | 0.72 (0.58, 0.90) | 0.68 (0.54, 0.86) | 0.69 (0.53, 0.90) | | 0.65 (0.49, 0.85) |  |
| 3+ | 0.90 (0.71, 1.14) | 0.77 (0.60, 0.99) | 0.89 (0.68, 1.18) | | 0.77 (0.57, 1.03) |  |
| Degree level educational attainment | 1.02 (0.87, 1.19) | 0.96 (0.81, 1.13) | 1.06 (0.88, 1.28) | | 0.98 (0.81, 1.19) |  |
| Mother history of eczema | 1.61 (1.40, 1.85) | 1.48 (1.27, 1.73) | 1.49 (1.24, 1.78) | | 1.34 (1.10, 1.64) |  |
| Antibiotics prescribed in first trimester | 1.26 (1.06, 1.49) | 1.21 (1.00, 1.45) | 1.14 (0.92, 1.41) | | 1.13 (0.89, 1.43) |  |
| Antibiotics prescribed in second trimester | 1.19 (1.00, 1.41) | 1.04 (0.86, 1.27) | 1.14 (0.92, 1.41) | | 0.99 (0.77, 1.26) |  |
| Antibiotics prescribed in third trimester | 1.04 (0.85, 1.27) | 0.85 (0.67, 1.08) | 1.04 (0.85, 1.27) | | 0.72 (0.53, 0.98) |  |
| Female sex of child | 0.97 (0.85, 1.11) | 0.99 (0.85, 1.14) | 0.94 (0.80, 1.10) | | 0.95 (0.80, 1.14) |  |
| Birthweight | 1.00 (1.00, 1.00) | 1.00 (1.00, 1.00) | 1.00 (1.00, 1.00) | | 1.00 (1.00, 1.00) |  |
| Child ever breastfed | 0.97 (0.84, 1.11) | 0.98 (0.84, 1.14) | 1.03 (0.87, 1.21) | | 1.00 (0.83, 1.21) |  |
| Antibiotics prescribed to child before 2 years of age | 1.77 (1.50, 2.09) | 1.44 (1.21, 1.73) | 1.97 (1.61, 2.42) | | 1.68 (1.34, 2.10) |  |
| Month/year of birth | 1.00 (0.99, 1.00) | 1.00 (0.99, 1.00) | 1.00 (0.99, 1.00) | | 1.00 (0.99, 1.00) |  |

Table S8: Risk ratios (IRR) with 95% confidence intervals (95% CI) for the association between timing of intrapartum antibiotic prophylaxis administration and allergic rhinitis diagnosed between by 5 and 2 years. Complete case analysis.

|  | Allergic rhinitis diagnosed by 5 years | | Allergic rhinitis diagnosed by 2 years | |
| --- | --- | --- | --- | --- |
|  | Unadjusted | Adjusted | Unadjusted | Adjusted |
| Prophylactic antibiotics administered before CS | 0.81 (0.55, 1.19) | 1.41 (0.69, 2.90) | 0.87 (0.49, 1.53) | 1.74 (0.68, 4.47) |
| Ethnicity |  |  |  |  |
| White British | 1.00 | 1.00 | 1.00 | 1.00 |
| Pakistani | 1.90 (1.34, 2.70) | 2.31 (1.51, 3.55) | 1.23 (0.76, 2.00) | 1.40 (0.77, 2.55) |
| Other ethnicity | 1.61 (1.06, 2.46) | 2.01 (1.24, 3.28) | 0.99 (0.52, 1.84) | 1.56 (0.81, 3.02) |
| Smoked during pregnancy | 0.71 (0.43, 1.19) | 1.02 (0.60, 1.74) | 0.88 (0.44, 1.75) | 0.90 (0.42, 1.92) |
| Number of children aged <16 years living in household |  |  |  |  |
| None | 1.00 | 1.00 | 1.00 | 1.00 |
| 1 | 1.15 (0.80, 1.66) | 1.06 (0.73, 1.53) | 1.10 (0.63, 1.90) | 1.02 (0.59, 1.76) |
| 2 | 0.79 (0.49, 1.26) | 0.63 (0.39, 1.03) | 0.87 (0.44, 1.70) | 0.75 (0.37, 1.51) |
| 3+ | 0.68 (0.38, 1.22) | 0.49 (0.27, 0.90) | 0.55 (0.21, 1.42) | 0.42 (0.16, 1.10) |
| Degree level educational attainment | 0.88 (0.70, 1.39) | 0.84 (0.59, 1.19) | 0.89 (0.53, 1.48) | 0.80 (0.47, 1.36) |
| Mother history of hay fever | 2.47 (1.86, 3.28) | 2.11 (1.52, 2.93) | 2.45 (1.59, 3.77) | 2.25 (1.38, 3.67) |
| Antibiotics prescribed in first trimester | 1.12 (0.77, 1.64) | 0.82 (0.52, 1.30) | 1.58 (0.94, 2.63) | 1.10 (0.60, 2.02) |
| Antibiotics prescribed in second trimester | 1.18 (0.82, 1.71) | 1.10 (0.72, 1.69) | 1.33 (0.77, 2.27) | 1.19 (0.65, 2.18) |
| Antibiotics prescribed in third trimester | 1.22 (0.81, 1.84) | 1.03 (0.63, 1.68) | 1.70 (0.87, 2.94) | 1.10 (0.55, 2.21) |
| Female sex of child | 0.86 (0.65, 1.15) | 0.82 (0.59, 1.13) | 1.02 (0.66, 1.57) | 1.07 (0.67, 1.71) |
| Birthweight (grams) | 1.00 (1.00, 1.00) | 1.00 (1.00, 1.00) | 1.00 (1.00, 1.00) | 1.00 (1.00, 1.00) |
| Child ever breastfed | 1.01 (0.76, 1.35) | 1.08 (0.78, 1.49) | 0.75 (0.49, 1.15) | 0.75 (0.47, 1.18) |
| Antibiotics prescribed to child before 2 years of age | 1.54 (1.11, 2.14) | 1.42 (0.99, 2.08) | 1.32 (0.82, 2.12) | 1.08 (0.64, 1.83) |
| Month/year of birth | 1.00 (0.99, 1.00) | 0.99 (0.99, 1.00) | 1.00 (0.99, 1.00) | 0.99 (0.98, 1.00) |

Table S9: Results of sensitivity analysis excluding children born March 2018 onwards who were 0-2 years during COVID-19 restrictions.

| N=2814 | Adjusted risk ratio, 95% CI, p | |
| --- | --- | --- |
|  | At 5 years | At 2 years |
| Asthma/wheeze | 0.99 (0.55 – 1.80), 0.98 | 1.55 (0.82 – 2.93), 0.17 |
| Eczema | 0.94 (0.67 – 1.33), 0.74 | 1.00 (0.67 – 1.50), 1.00 |
| Allergic rhinitis | 1.20 (0.61 – 2.36), 0.60 | 1.50 (0.62 – 3.60), 0.37 |
|  |  |  |
